# Revision of the systematics of the Polystomoidinae (Platyhelminthes, Monogenea, Polystomatidae) with redefinition of *Polystomoides* Ward, 1917 and *Uteropolystomoides* Tinsley, 2017[Fn FN1]

**DOI:** 10.1051/parasite/2022056

**Published:** 2022-12-16

**Authors:** Amira Chaabane, Louis Du Preez, Gerald R. Johnston, Olivier Verneau

**Affiliations:** 1 Unit for Environmental Sciences and Management, North-West University, Potchefstroom Campus Private Bag X6001 Potchefstroom 2520 South Africa; 2 South African Institute for Aquatic Biodiversity Private Bag 1015 Makhanda 6140 South Africa; 3 Department of Natural Sciences, Santa Fe College Gainesville Florida 32606 USA; 4 University of Perpignan Via Domitia, Centre de Formation et de Recherche sur les Environnements Méditerranéens, UMR 5110 66860 Perpignan France; 5 CNRS, Centre de Formation et de Recherche sur les Environnements Méditerranéens, UMR 5110 66860 Perpignan France

**Keywords:** Polystomatidae, *Neopolystoma*, *Polystomoides*, *Uteropolystomoides*, Classification, Systematics

## Abstract

Polystomatids are platyhelminth parasites that infect mainly amphibians and freshwater turtles. For more than seven decades, chelonian polystomes were classified into three genera according to the number of hamuli, *i.e.* absent for *Neopolystoma*, one pair for *Polystomoidella* and two pairs for *Polystomoides*. Following re-examination of morphological characters, seven new genera were erected the past six years, namely *Apaloneotrema*, *Aussietrema*, *Fornixtrema*, *Manotrema*, *Pleurodirotrema*, *Uropolystomoides* and *Uteropolystomoides*. However, the polyphyly of *Neopolystoma* and *Polystomoides* on the one hand, and the nested position of *Uteropolystomoides* within a clade encompassing all *Neopolystom*a and *Polystomoides* spp. on the other, still raised questions about the validity of these genera. We therefore re-examined several types, paratypes and voucher specimens, and investigated the molecular phylogeny of polystomes sampled from the oral cavity of North American turtles to re-evaluate their systematic status. We show that all *Polystomoides* Ward, 1917, *sensu* Du Preez et al., 2022, *Neopolystoma* Price, 1939, *sensu* Du Preez et al., 2022 and *Uteropolystomoides* Tinsley, 2017 species, display vaginae that are peripheral and extend well beyond the intestine. We thus reassign all species of the clade to *Polystomoides* and propose nine new combinations; however, although *Uteropolystomoides* is nested within this clade, based on its unique morphological features, we propose to keep it as a valid taxon. *Polystomoides* as redefined herein groups all polystome species infecting either the oral cavity or the urinary bladder of cryptodires, with peripheral vaginae and with or without two pairs of small hamuli. *Uteropolystomoides nelsoni* (Du Preez & Van Rooyen 2015), originally described from *Pseudemys nelsoni* Carr is now regarded as *Uteropolystomoides multifalx* (Stunkard, 1924) n. comb. infecting three distinct *Pseudemys* species of North America.

## Abstract (fr)

Les Polystomatidés sont des plathelminthes parasites qui infestent principalement les amphibiens et les tortues d’eau douce. Pendant plus de sept décennies, les polystomes de chéloniens ont été classés en trois genres selon le nombre d’hamuli, absents pour *Neopolystoma*, une paire pour *Polystomoidella* et deux paires pour *Polystomoides*. Suite au réexamen des caractères morphologiques, sept nouveaux genres ont été érigés ces six dernières années, à savoir *Apaloneotrema*, *Aussietrema*, *Fornixtrema*, *Manotrema*, *Pleurodirotrema*, *Uropolystomoides* et *Uteropolystomoides*. Cependant, la polyphylie de *Neopolystoma* et *Polystomoides* d’une part, et la position imbriquée d’*Uteropolystomoides* au sein d’un clade englobant toutes les espèces de *Neopolystoma* et *Polystomoides* d’autre part, soulèvent encore des questions sur la validité de ces trois genres. Nous avons donc réexaminé plusieurs types, paratypes et vouchers et étudié la phylogénie moléculaire de polystomes prélevés dans la cavité buccale de tortues d’Amérique du Nord pour réévaluer leur statut systématique. Nous montrons que toutes les espèces de *Polystomoides* Ward, 1917, *sensu* Du Preez et al., 2022, *Neopolystoma* Price, 1939, *sensu* Du Preez et al., 2022 et *Uteropolystomoides* Tinsley, 2017, présentent des vagins périphériques qui s’étendent bien au-delà de l’intestin. Nous réattribuons ainsi toutes les espèces du clade à *Polystomoides* et proposons neuf nouvelles combinaisons; cependant, nous proposons de conserver *Uteropolystomoides* sur la base de ses caractéristiques morphologiques exceptionnelles, bien que son espèce soit imbriquée au sein de ce clade. *Polystomoides* tel que redéfini ici regroupe toutes les espèces de polystomes infectant soit la cavité buccale, soit la vessie des cryptodires, avec des vagins périphériques, et deux paires de petits hamuli ou sans hamuli. *Uteropolystomoides nelsoni* (Du Preez & Van Rooyen 2015), l’unique espèce décrite à l’origine à partir de *Pseudemys nelsoni* Carr est maintenant considérée comme *Uteropolystomoides multifalx* (Stunkard, 1924) n. comb., qui infecte trois espèces distinctes de *Pseudemys* d’Amérique du Nord.

## Introduction

The Neodermata, a clade comprising only parasitic platyhelminths, contains three well-defined groups of flatworms, the Digenea, the Cestoda and the Monogenea. While the monophyly of the Monogenea is still being debated [23, 27, 31, 34], the monophyly of the two subclasses Polyonchoinea and Heteronchoinea has been widely accepted [3–5, 24–26, 29, 31]. Monogeneans of both subclasses are mainly ectoparasites of gills and skin of Chondrichthyes and Actinopterygii fishes, which may account for more than 25,000 species [9, 47]. However, fewer than 250 monogenean species deviated from the norm as they are parasites of semi-aquatic tetrapods, mainly amphibians and chelonians. They are classified into three families of the Polyonchoinea, namely the Gyrodactylidae, the Lagarocotylidae and the Iagotrematidae, and into a single family of the Heteronchoinea, the Polystomatidae sensu Sinnappah et al. [37]. The Polystomatidae comprises just more than 200 species, infecting anurans, salamanders and caecilians of the Amphibia; freshwater turtles of the Testudines; the common hippopotamus, *Hippopotamus amphibius* Linnaeus of the Mammalia; but also a fish, *i.e.* the Australian lungfish, *Neoceratodus forsteri* Krefft of the Dipnoi. Polystome species are classified into 32 genera, of which 20 occur specifically within amphibian hosts, 10 are recognized in chelonians, and one each are reported from the common hippopotamus and Australian lungfish, respectively.

Polystomes of frogs and chelonians were first described as *Polystoma* Zeder, 1800, and more than a century later a new subgenus *Polystomoides* Ward, 1917 was created to account for chelonian polystomes. *Polystomoides*, being found in the mouth, esophagus, nasal cavities or urinary bladder of its host, was described as having a haptor with two pairs of large hooks, the outer pair being larger than the inner one, a single testis, a short uterus containing usually a single egg and vitellaria extending into the posterior part of the body. Vaginae and eyes are absent in adults. *Polystomoides* was raised later to genus rank by Ozaki [32] who pointed out the absence of a uterus. Besides *Polystomoides*, Price [36] created two new genera for chelonian polystomes, namely *Polystomoidella* Price, 1939 being found in the urinary bladder of its host and differing from *Polystomoides* by having a single pair of large haptoral hooks, and *Neopolystoma* Price, 1939, being found in the urinary bladder and nostrils of its host and differing from *Polystomoides* and *Polystomoidella* by the absence of large haptoral hooks. Strelkov [38] first reported *Neopolystoma* from conjunctival sacs of turtles. Tinsley and Tinsley [43], based on phylogenetic studies by Héritier et al. [11], created a new genus *Uropolystomoides* Tinsley & Tinsley 2016 to account for all *Polystomoides* species occurring in the urinary bladder of their African, Asian, and Australian hosts. *Uropolystomoides* spp. differ from *Polystomoides* spp. of the oral cavity by the size of hamulus 1, being always bigger than the sucker diameter, which was originally mentioned in Knoepffler and Combes [18]. Tinsley [42], following the description of *Polystomoides nelsoni* Du Preez & Van Rooyen 2015, created *Uteropolystomoides* Tinsley 2017 to account for this unique species. *Uteropolystomoides nelsoni* (Du Preez & Van Rooyen 2015) differs from species of *Polystomoides* sensu Tinsley and Tinsley [43] by the presence of a uterus containing several eggs but also by a massive genital bulb encompassing a great number of genital spines. Du Preez and Verneau [8], based on the most comprehensive phylogeny of chelonian polystomes, created three new genera to account for all polystomes of the conjunctival sacs, namely *Aussietrema* Du Preez & Verneau 2020, *Fornixtrema* Du Preez & Verneau 2020, and *Apaloneotrema* Du Preez & Verneau 2020. *Aussietrema* is mainly characterized by a spherical ovary and egg, *Fornixtrema* by a separate egg-cell-maturation-chamber and fusiform to diamond-shaped egg with acute tips, and *Apaloneotrema* by a large fusiform egg with rounded tips. Finally, Du Preez et al. [7], following a revision of South American and Australian polystomes infecting specifically turtles of the Pleurodira suborder, described two new genera that are both restricted to South America and Australia, respectively. Though these two genera share vaginae that are latero-ventral and positioned in line with the anterior margin of testis, *Manotrema* Du Preez, Domingues & Verneau 2022 of South American pleurodires differs from *Pleurodirotrema* Du Preez, Domingues & Verneau 2022 of Australian pleurodires by the presence of two pairs of small hamuli with very deep cuts between handle and guard and a haptor with deep incisions between suckers.

While Bayesian trees inferred from phylogenetic analyses of the four concatenated genes 12S, 18S, 28S and COI [8, 11] indicate that the two genera, *i.e. Polystomoides* sensu Du Preez et al. [7] and *Neopolystoma* sensu Du Preez et al. [7], are each polyphyletic, all *Polystomoides* and *Neopolystoma* species fall into a robust lineage, including *U. nelsoni* of *Pseudemys nelsoni* Carr. Therefore, one may question the possibility of finding specific morphological characters for this clade. In this paper, we studied polystome samples collected from North American chelonians, type and paratype slides borrowed from the Parasitic Worm Collection, National Museum, Bloemfontein, South Africa, and voucher slides stored in the private collection of the second author (LdP) to revise the classification of these two genera. We also investigated the molecular phylogeny of polystomes sampled from the oral cavity of North American turtles, including specimens of *Polystomoides multifalx* (Stunkard, 1924) collected from *Pseudemys floridana* (Le Conte) and *Pseudemys concinna* (Le Conte) of Florida, in order to determine the validity of the genus *Uteropolystomoides*.

## Materials and methods

### Ethics

Ethical clearance for this study was obtained from the North-West University Animal Care ethics committee (Ethical clearance no. NWU-00256-17A5).

### Turtle sampling and polystome collection

The fieldwork procedures used to collect freshwater turtles were detailed in Du Preez and Verneau [8]. To summarize, turtles were captured in a number of water bodies in North Carolina and Florida, USA using baited traps that were left overnight (Table 1). Captured animals were kept individually in plastic containers at room temperature for two to three days and screened on a daily basis for the presence of polystome eggs following the procedure detailed in Verneau et al. [45]. Polystome eggs collected were preserved in ethanol 75% for further molecular analyses. Depending on the intensity of infection, based on the number of eggs released per host individual, a few animals were euthanized with a lethal injection of a concentrated buffered MS222 (ethyl-4-aminobenzoate) solution. They were then dissected and polystomes were retrieved from the urinary bladder, oral cavity, and/or conjunctival sacs. Polystomes were removed according to the procedure reported in Du Preez and Verneau [8].

**Table 1:** List of North American turtle species investigated for polystomes in 2018–2019, with sampling localities and their GPS coordinates, prevalence of infection, infection site of polystomes, and total number of worms collected.

Host species	State	Year	Locality	GPS coordinates	No. of turtles infected / examined	Type of Eggs	No. of dissected turtles	Infection site of polystomes	No. of worms collected
*Chrysemys picta*	North Carolina	2018	Fountain twin pond	35,499030 N, −80,863681 W	0/10		0		
*C. picta*	North Carolina	2018	Small Griffith pond	35,501874 N,	2/9	Round &	2	Oral cavity &	6
				−80,855274 W		long		conj. sacs	4
*C. picta*	North	2018	Big Griffith	35,502340 N,	0/1		0		
	Carolina		Pond	−80,856278 W					
*C. picta*	North	2018	Lake Norman	35,570090 N,	0/4		0		
	Carolina		Mooresville	−80,856500 W					
*C. picta*	North	2018	Norman’small	35,570824,	0/9		3	Oral cavity	2-1-0
	Carolina		Pond	−90,848196 W					
*C. picta*	North	2018	Mooresville	35,575493 N,	0/6		0		
	Carolina		golf course	−80,835494 W					
*C. picta*	North	2018	Carringan	35,604860,	0/2		0		
	Carolina		farm pond	−80,767886					
*Trachemys*	North	2018	Fountain twin	35,499030 N,	3/4	Round	1	Oral cavity	6
*scripta*	Carolina		Pond	−80,863681 W					
*T. scripta*	North	2018	Big Griffith	35,502340 N,	4/4	Round	1	Oral cavity	2
	Carolina		Pond	−80,856278 W					
*T. scripta*	North	2018	Lake Norman	35,570090 N,	3/5	Round	1	Oral cavity	3
	Carolina		Mooresville	−80,856500 W					
*T. scripta*	North	2018	Mooresville	35,575493 N,	12/17	Round &	3	Oral cavity &	14-8-2
	Carolina		golf course	−80,835494 W		long		conj. sacs	1-0-2
*T. s. scripta*	Florida	2018	Gainesville	29,670146 N,	4/9	Round &	3**	Oral cavity &	0-1-0
			Pond	−82,401368 W		long		urin. bladder &	0-2-0
								conj. sacs	1-0-0
*T. s. scripta*	Florida	2018	Hornsby Spring	29,850280 N,	2/3	Round	2**	Urin. bladder	3-1
				−82,593300 W					
*T. s. scripta*	Florida	2018	Santa Fe College	29,683781 N,	1/2	Round	0		
			campus pond 1	−82,434605 W					
*T. s. scripta*	Florida	2018	Santa Fe College	29,683781N,	0/2	0			
			campus pond 3	−82,434618 W					
*T. s. scripta*	Florida	2018	Ichetucknee	29,969430 N,	1/7	Round	0		
			River	−82,785930 W					
*T. s. scripta*	Florida	2018	Quail Heights	30,166279 N,	8/15	Round &	2**	Oral cavity &	2-2
			golf course	−82,673098 W		long		conj. sacs	0-1
*T. s. scripta*	Florida	2018	Hornsby Spring	29,850239 N,	0/1		0		
				−82,893722 W					
*T. s. scripta*	Florida	2018	Deer Run	29,716736 N,			2**	Oral cavity	0-3
			Gainesville	−82,297859 W					
*Pseudemys*	Florida	2018	Ichetucknee	29,969430 N,	6/13	Round	3*	Oral cavity	1-1-1
*concinna*			River	−82,785930 W					
*P. concinna*	Florida	2018	Hornsby Spring	29,850239 N,	3/9	Round &	1	Oral cavity &	3
				−82,893722 W		long		conj. sacs	7
*P. concinna*	Florida	2018	Ichnetucknee	29,952778 N,			1**	Oral cavity &	3
			Bridge	−82,679350 W				conj. sacs	1
*P. concinna*	Florida	2019	Santa Fe River	29,833525 N,	5/11	Round	1	Oral cavity	1
				−82,679350 W					
*Pseudemys*	Florida	2018	Deer Run	29,716736 N,			4**	Oral cavity &	4-0-0-3
*nelsoni*			Gainesville	−82,297859 W				conj. sacs	1-4-0-0
*Pseudemys*	Florida	2018	Hornsby Spring	29,850239 N,	1/1	Round	1	Oral cavity	1
*floridana*				−82,893722 W					
*P. floridana*	Florida	2018	Deer Run	29,716736 N,			1**		0
			Gainesville	–82,297859 W					
*P. floridana*	North	2018	Mooresville	35,575493 N,	1/1	Round	1**	Oral cavity	1
	Carolina		golf course	–80,835494 W					
*Pseudemys*	Florida	2018	Hornsby Spring	29,850239 N,	1/1	Round &	1	Oral cavity &	1
*peninsularis*				–82,893722 W		long		conj. sacs	2
*P. peninsularis*	Florida	2018	Deer Run	29,716736 N,			1**	Conj. sacs	9
			Gainesville	–82,297859 W					
*Apalone ferox*	Florida	2018	Gainesville	29,670146 N,	0/2		0		
			pond	–82,401368 W					
*A. ferox*	Florida	2018	Gainesville	29,7 N,			6**	Oral cavity &	11-0-6-3-1-1
				−82,3 W				conj. sacs	4-14-0-0-0-0
*A. ferox*	Florida	2018	Spanish Spring	28,943611 N,			1**	Oral cavity &	11
				−81,950833 W				conj. sacs	4

Abbreviations used: Conj. sacs = Conjunctival sacs; Urin. bladder = Urinary bladder.

* refers to infected turtles that were surveyed for polystomes using a non-destructive approach with swabs rotated in the throat.

** refers to road kills that were frozen until dissection.

### Collection of polystomes using a non-lethal method

**Figure 1:**
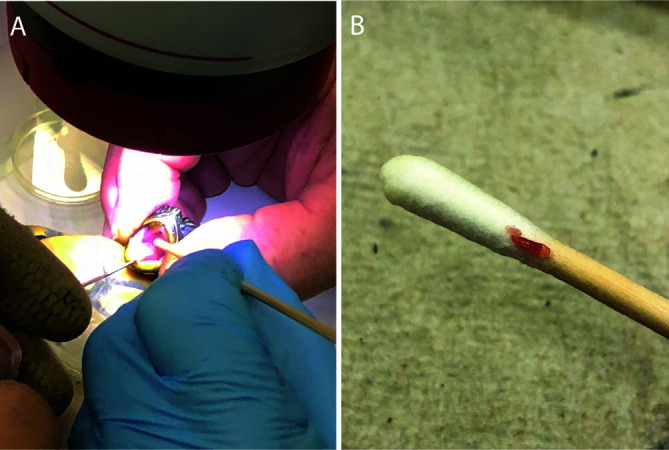
A: Non-lethal procedure for retrieving a polystome from the pharyngeal pouches of a freshwater turtle; B: polystome collected on wooden stem cotton swab.

Because killing of animals collected from the Ichetucknee River in Ichetucknee Springs State Park of Florida was not allowed, specimens of *P. concinna* that released polystome eggs were examined by swabbing the mouth and pharyngeal pouches. The turtle was held with the head facing upwards and the mouth held open with a small hook made from wire (Fig. 1A). A dry 120 mm wooden stem cotton swab was gently lowered down the mouth into the pharyngeal region while slowly rotating the swab. The technique was successful, and three parasites were retrieved from three distinct specimens (Fig. 1B, Table 1) with no adverse effect on the hosts. Parasites were heat killed and stored for further analysis. Some were fixed slightly flattened under coverslip pressure, while others were fixed directly either in 10% neutral buffered formalin for permanent mounts, in Bouin’s fixative [15] for histology or in molecular grade 70% ethanol for genetics.

### Morphological analyses

In 2004, LdP visited the United States National Parasite Collection in Beltsville, Maryland for a research visit and studied the entire polystome collection. A loan of voucher and paratype specimens was approved and specimens were studied and photographed in South Africa. Type and paratype slides borrowed from the Parasitic Worm Collection, National Museum, Bloemfontein, South Africa were also examined as well as voucher slides stored in the second author’s collections (Table 2). All slides were of whole-mounted stained specimens. While the main focus was on species belonging to *Neopolystoma*, *Polystomoides*, and *Uteropolystomoide*s, representatives of *Fornixtrema* and *Uropolystomoides* were also examined. Polystomes infecting *P. nelsoni* and *P. concinna* were morphologically examined, measured, and photographed using a Nikon AZ100M microscope (Nikon, Netherlands) fitted with 0.5X, 1X and 4X objectives as well as a Nikon U3 digital camera. Measurements were captured using the Nikon NIS software. Small features were examined, measured, and photographed using a Zeiss Imager Axio10 compound microscope (Zeiss, Germany) fitted with a Zeiss Axio cam 305 camera (Zeiss, Germany) and Zeiss Zen Blue elements (Zeiss, Germany) software. Measurements were based on ten specimens each from *P. nelsoni* and *P. concinna*, all collected near Gainesville, Alachua County, FL, USA. Morphological examination focussed on body size, relative size of the haptor, genital bulb diameter, number of genital spines, position of the vaginae in relation to body width and length, position of ovary, position of testis, presence and size of hamuli and haptoral sucker diameter.

**Table 2:** List of polystome species examined by microscopy with their host species, geographical area, infection site and accession numbers.

Polystome species	Host species	Locality	GPS coordinates	Infection site	Polystome specimens (Accession number)
*Neopolystoma cayensis*	*Rhinoclemmys punctularia*	Cayenne, French Guiana	4,87082 N, −52,33678 W	Urinary bladder	NMB P394 Holotype (=PL120414E10)
					NMB P395–P402 Paratypes (=PL120414E2–PL120414E9)
					NMB P403 Paratype (=PL120415A8)
*Neopolystoma orbiculare*	*Chrysemys picta*	Davidson, North Carolina, USA	35,501874 N,	Urinary bladder	PL150729E4
			−80,855274 W		PL150729E5–PL150729E6
*Polystomoides asiaticus*	*Cuora amboinensis*	Kuala Lumpur, Malaysia	Unknown (From pet shop)	Oral cavity	PL990512K2
					PL980316B2
*Polystomoides stunkardi*	*Pseudemys concinna*	Ichetucknee spring, Florida, USA	29,969430 N, −82,785930 W	Oral cavity	PL180716C1–PL180716C2
					PL180719F20–PL180719F22
					PL180719F24–PL180719E26
					PL180719B2–PL180719B3
					PL180720A1
*Polystomoides oris*	*C. picta*	Davidson,	35,501874 N, −80,855274 W	Oral cavity	PL150729D2
		North Carolina, USA			PL150729D3
					PL150729D4
*Polystomoides scriptanus*	*Trachemys scripta*	Davidson,	35,461806 N, −80,802833 W	Oral cavity	NMB P434
		North Carolina, USA			(=PL150728B2)
					NMB P435
					(=PL150728B3)
*Polystomoides soredensis*	*Emys orbicularis*	Sorède, France	42,515556 N, 2,957694 E	Oral cavity	PL060528E3
					PL060528E4
					PL060528E6
					PL060528E8
					PL060528E10
					(=NMB P429–433)
*Uteropolystomoides nelsoni*	*Pseudemys nelsoni*	Gainesville, Florida, USA	29,725278 N, −82,417778 W	Oral cavity	NMB P380 Holotype (=PL040625C9)
					NMB P381–P389 Paratypes
					(=PL040625C2–PL040625C11)
*Uropolystomoides malayi*	*C. amboinensis*	Kuala Lumpur, Malaysia	Unknown (From pet shop)	Urinary bladder	PL980318C1
					PL990513C1
*Uropolystomoides* sp.	*Pelomedusa subrufa*	Benin City, Nigeria	Unknown (From street market)	Urinary bladder	PL070503A2–PL070503A4
					PL070503B2–PL070503B4
					PL070504A2–PL070504A3
					PL070504C1–PL070504C3
*Fornixtrema grossi*	*Pseudemys floridana*	Gainesville, Florida, USA	29,725278 N, −82,417778 W	Conjunctival sacs	NMB P341
					Holotype
					(=PL040612B1)
					NMB P342–P343 Paratypes
					(=PL040612B2–PL040612B3)
*Fornixtrema guianensis*	*R. punctularia*	Cayenne, French Guiana,	4,87082 N, −52,33678 W	Conjunctival sacs	NMB P404 Holotype
					(=PL120421A3)
					NMB P405–P406 Paratypes
					(=PL120421C3–PL120421C4)
*Fornixtrema liewi*	*C. amboinensis*	Kuala Lumpur, Malaysia	3,128889 N, 101,655278 E	Conjunctival sacs	NMB P222–P223
					(=PL990513B1–PL990513B2)
					NMB P224
					(=PL980312B2)
					NMB P225
					(=PL990506C2)
					NMB P226–P227
					(=PL980411A1–PL980411A2)
*Fornixtrema scorpioides*	*Kinosternon scorpioides*	Roura, French Guiana	4,66997 N, −52,30560 W	Conjunctival sacs	PL120415C2–PL120415C4

*Fornixtrema* sp.	*Pseudemys concinna*	Hornsby Spring, Florida, USA	29,850239 N, −82,893722 W	Conjunctival sacs	PL180719E1–PL180719E4
					PL180719G2–PL180719G7

### Molecular experiments

DNA extractions were performed with 150 μL of Chelex 10% and Proteinase K 1 mg/mL, following the protocol reported in Héritier et al. [11], from several eggs and worms collected from distinct host species and areas of North Carolina and Florida (Table 3). For the PCR, we followed the amplification procedure of Héritier et al. [11] for the two genes of interest COI and 28S. COI was amplified in one round, either with primers L-CO1p/H-Cox1p2 or L-CO1p/H-Cox1R whose sequences are reported in Littlewood et al. [22] and Héritier et al. [11]. The partial 28SrRNA gene was, however, amplified in two rounds with the combination of primers LSU5′/IR16 and IF15/LSU3′ whose sequences are reported in Verneau et al. [44] and Héritier et al. [11]. The procedure we followed for gene amplification was identical regardless of the combination of primers and gene of interest: one initial step of 5′ at 95 °C for long denaturation; 30 cycles of 1′ at 95 °C for denaturation, 1′ at 48 °C for annealing and 1′ at 72 °C for elongation; one final step of 10′ at 72 °C for terminal elongation. PCR reactions were run twice and independently in a final volume of 25 μL comprising Buffer 1x, MgCl_2_ 1.5 mM, dNTPs 0.2 mM, primers 0.4 mM, GoTaq Polymerase 0.75 unit (Promega, France) and DNA (2 μL). PCR products were then pooled and sent to GenoScreen (Lille, France) for purification and sequencing with their respective forward and reverse PCR primers. Finally, we used Geneious software (Saint Joseph, MO, USA) to check chromatograms, and to read and edit resulting sequences. New sequences were deposited in GenBank with accession numbers OP784895, OP793140 to OP793161 and OP793434 to OP793461 for COI, and OP795734 to OP795746 and OP795805 to OP795807 for 28S.

**Table 3:** List of turtle specimens collected in the USA in 2018 from which polystome worms and/or eggs were investigated for partial COI and 28S.

Host species	Locality	Host field no.	Par. tiss.	Par. field no.	Inf. site	DNA no.	COI hap. no.	G.B.A. no.	28S hap. no.	G.B.A. no.	Parasite species
	**North Carolina**										
*T. scripta*	Fountain twin pond	RL180703C1	1 egg	3C1	Oral cav.	MiAE97	H149, 372 bp	OP793140			*P. soredensis*
*T. scripta*	Fountain twin pond	RL180705A1	1 worm	PL180707L5	Oral cav.	MiAG93	H149, 372 bp	OP793141			*P. soredensis*
*T. scripta*	Big Griffith pond	RL180704C1	1 egg	4C1	Oral cav.	MiAE102	H64, 396 bp	OP793142			*P. soredensis*
*T. scripta*	Big Griffith pond	RL180705C1	1 egg	5C1	Oral cav.	MiAE135	H64, 333 bp	OP793143			*P. soredensis*
*T. scripta*	Big Griffith pond	RL180705C2	1 egg	5C2	Oral cav.	MiAE132	H149, 363 bp	OP793144			*P. soredensis*
*T. scripta*	Big Griffith pond	RL180705C3	1 egg	5C3	Oral cav.	MiAE138	H64, 385 bp	OP793145			*P. soredensis*
*T. scripta*	Mooresville golf course	RL180704K5	1 egg	4K5	Oral cav.	MiAE114	H149, 372 bp	OP793146			*P. soredensis*
*T. scripta*	Mooresville golf course	RL180704K6	1 egg	4K6	Oral cav.	MiAE117	H64, 397 bp	OP793147			*P. soredensis*
*T. scripta*	Mooresville golf course	RL180704K7	1 egg	4K7	Oral cav.	MiAE120	H150, 372 bp	OP793148			*P. soredensis*
*T. scripta*	Mooresville golf course	RL180705J2	1 worm	PL180706A1	Oral cav.	MiAG19	H150, 367 bp	OP793149	Hnuc7, 964 bp	OP795734	*P. soredensis*
*T. scripta*	Mooresville golf course	RL180705J2	1 worm	PL180706A2	Oral cav.	MiAG21	H149, 394 bp	OP793150			*P. soredensis*
*T. scripta*	Mooresville golf course	RL180705J2	1 egg	5J2	Oral cav.	MiAE149	H64, 396 bp	OP793151			*P. soredensis*
*T. scripta*	Mooresville golf course	RL180705J2	1 egg	5J2	Oral cav.	MiAE151	H64, 387 bp	OP793152			*P. soredensis*
*T. scripta*	Mooresville golf course	RL180705J2	1 egg	5J2	Oral cav.	MiAE152	H150, 372 bp	OP793153			*P. soredensis*
*T. scripta*	Mooresville golf course	RL180706G1	1 egg	6G1	Oral cav.	MiAE155	H150, 372 bp	OP793154			*P. soredensis*
*T. scripta*	Mooresville golf course	RL180704K3	1 egg	4K3	Oral cav.	MiAE109	H158, 323 bp	OP793155			*Polystomoides* sp2
*T. scripta*	Mooresville golf course	RL180704K5	1 worm	PL180707D3	Oral cav.	MiAG64	H157, 396 bp	OP793156	Hnuc36, 1366 bp	OP795735	*Polystomoides* sp2
*T. scripta*	Mooresville golf course	RL180704K5	1 worm	PL180707D1	Oral cav.	MiAG65	H157, 396 bp	OP793157	Hnuc36, 927 bp	OP795736	*Polystomoides* sp2
*T. scripta*	Mooresville golf course	RL180704K10	1 worm	PL180707E1	Oral cav.	MiAG87	H157, 396 bp	OP793158	Hnuc36, 1353 bp	OP795737	*Polystomoides* sp2
*T. scripta*	Mooresville golf course	RL180704K10	1 egg	4K10	Oral cav.	MiAE123	H157, 346 bp	OP793159			*Polystomoides* sp2
*T. scripta*	Lake Norman Mooresville	RL180705F1	1 egg	5F1	Oral cav.	MiAE141	H152, 372 bp	OP793160			*P. soredensis*
*T. scripta*	Lake Norman Mooresville	RL180706D2	1 egg	6D2	Oral cav.	MiAE142	H64, 366 bp	OP784895			*P. soredensis*
*C. picta*	Small Griffith pond	RL180703A6	1 worm	PL180707K4	Oral cav.	MiAG10	H99, 372 bp	OP793161			*P. oris*
*C. picta*	Small Griffith pond	RL180703A6	1 worm	PL180707K5	Oral cav.	MiAG11	H99, 345 bp	OP793162			*P. oris*
*C. picta*	Small Griffith pond	RL180703A6	1 egg	3A6	Oral cav.	MiAE145	H99, 344 bp	OP793163			*P. oris*
*C. picta*	Small Griffith pond	RL180704B1	1 egg	4B1	Oral cav.	MiAE147	H99, 372 bp	OP793164			*P. oris*
*C. picta*	Norman’small pond	RL180704G1	1 worm	PL180704A1	Oral cav.	MiAG9	H156, 372 bp	OP793165			*P. oris*
	**Florida**										
*T. scripta*	Gainesville pond	RL180709B1	1 worm	PL180716F1	Oral cav.	MiAG67	H154, 372 bp	OP793434	Hnuc21, 1341 bp	OP795738	*P. scriptanus*
*T. scripta*	Gainesville pond	RL180711B5	1 egg	11B5	Oral cav.	MiAE19	H155, 396 bp	OP793435			*P. scriptanus*
*T. scripta*	Quial Heights golf course	RL180713C3	1 worm	PL180716K1	Oral cav.	MiAG66	H151, 396 bp	OP793436	Hnuc7, 964 bp	OP795739	*P. soredensis*
*T. scripta*	Quial Heights golf course	RL180713C9	1 egg	13C9	Oral cav.	MiAE43	H151, 387 bp	OP793437			*P. soredensis*
*T. scripta*	Quial Heights golf course	RL180713C1	1 worm	PL180718I1	Oral cav.	MiAG68	H153, 396 bp	OP793438	Hnuc21, 964 bp	OP795740	*P. scriptanus*
*T. scripta*	Quial Heights golf course	RL180713C1	1 egg	13C1	Oral cav.	MiAE23	H153, 370 bp	OP793439			*P. scriptanus*
*T. scripta*	Quial Heights golf course	RL180713C3	1 egg	13C3	Oral cav.	MiAE30	H153, 396 bp	OP793440			*P. scriptanus*
*T. scripta*	Quial Heights golf course	RL180713C5	1 egg	13C5	Oral cav.	MiAE37	H153, 396 bp	OP793441			*P. scriptanus*
*T. scripta*	Quial Heights golf course	RL180713C7	1 egg	13C7	Oral cav.	MiAE40	H153, 372 bp	OP793442	Hnuc21, 964 bp	OP795741	*P. scriptanus*
*P. peninsularis*	Hornsby Spring	RL180715B1	1 egg	15B1	Oral cav.	MiAE87	H153, 372 bp	OP793443	Hnuc21, 946 bp	OP795742	*P. scriptanus*
*P. peninsularis*	Hornsby Spring	RL180715B1	1 egg	15B1	Oral cav.	MiAE88	H153, 396 bp	OP793444			*P. scriptanus*
*P. peninsularis*	Hornsby Spring	RL180715B1	1 egg	15B1	Oral cav.	MiAE89	H153, 387 bp	OP793445			*P. scriptanus*
*P. concinna*	Ichetucknee bridge	RL180719A1	1 worm	PL180719B1	Oral cav.	MiAG82	H148, 396 bp	OP793446			*P. multifalx*
*P. concinna*	Ichetucknee River	RL180711A1	1 egg	11A1	Oral cav.	MiAE49	H145, 372 bp	OP793447	Hnuc20, 960 bp	OP795743	*P. multifalx*
*P. concinna*	Ichetucknee River	RL180711A1	1 egg	11A1	Oral cav.	MiAE50	H145, 387 bp	OP793448			*P. multifalx*
*P. concinna*	Ichetucknee River	RL180711A2	1 egg	11A2	Oral cav.	MiAE53	H146, 377 bp	OP793449			*P. multifalx*
*P. concinna*	Ichetucknee River	RL180711A5	1 egg	11A5	Oral cav.	MiAE56	H146, 372 bp	OP793450			*P. multifalx*
*P. concinna*	Ichetucknee River	RL180711A5	1 egg	11A5	Oral cav.	MiAE57	H147, 396 bp	OP793451			*P. multifalx*
*P. concinna*	Ichetucknee River	RL180711A6	1 egg	11A6	Oral cav.	MiAE60	H146, 387 bp	OP793452			*P. multifalx*
*P. concinna*	Ichetucknee River	RL180711A8	1 egg	11A8	Oral cav.	MiAE61	H147, 372 bp	OP793453			*P. multifalx*
*P. concinna*	Ichetucknee River	RL180711A1	1 worm	PL180716C1	Oral cav.	MiAG71	H146, 396 bp	OP793454	Hnuc20, 1366 bp	OP795744	*P. multifalx*
*P. concinna*	Hornsby Spring	RL180715D7	1 egg	15D7	Oral cav.	MiAE64	H148, 346 bp	OP793455			*P. multifalx*
*P. concinna*	Hornsby Spring	RL180715D9	1 worm	PL180719H2	Oral cav.	MiAG81	H146, 386 bp	OP793456			*P. multifalx*
*P. floridana*	Quial Heights golf course	RL180713D1	1 worm	PL180717L1	Oral cav.	MiAG34	H145, 394 bp	OP793457	Hnuc20, 964 bp	OP795745	*P. multifalx*
*P. floridana*	Quial Heights golf course	RL180713D1	1 egg	13D1	Oral cav.	MiAE83	H147, 372 bp	OP793458			*P. multifalx*
*P. floridana*	Quial Heights golf course	RL180713D1	1 egg	13D1	Oral cav.	MiAE84	H147, 372 bp	OP793459			*P. multifalx*
*A. ferox*	Spanish Spring	RL180712A1	1 egg	12A1	Oral cav.	MiAE95	H160, 336 bp	OP793460	Hnuc37, 1366 bp	OP795746	*N. rugosa*
*A. ferox*	Gainesville	RL180718D1	1 worm	PL180718F1	Oral cav.	MiAG78	H159, 372 bp	OP793461			*N. rugosa*

Abbreviations used: G.B.A. = GenBank Accession; hap. = haplotype; Inf. = infection; no. = number; Oral cav. = Oral cavity; Par. tiss. = Parasite tissue.

Underlined numbers indicate sequences used for analyses.

### Phylogenetic and distance analyses within polystomes of the pharyngeal cavity

New COI and 28S sequences, after primer trimming, were first aligned independently using Clustal W implemented in MEGA version 7 [19] under default parameters [41]. Only those characterizing polystomes of the oral cavity were kept at this stage. All these sequences were subsequently aligned with other COI and 28S sequences of distinct polystomes species retrieved from GenBank (Table 4). These sequences characterized polystomes of the oral cavity with the exception of *Fornixtrema palpebrae* (Strekov, 1950) of the conjunctival sacs and *Polystomoidella whartoni* Price, 1939 and *Uropolystomoides malayi* (Rohde, 1963) of the urinary bladder, that were used for outgroup comparisons after Du Preez and Verneau [8]. In the final COI and 28S alignments, when identical sequences were found from the sequencing of eggs and/or worm, a single sequence was kept for each distinct haplotype.

**Table 4:** List of polystome species retrieved from GenBank and investigated by phylogenetic analysis with references to their COI and 28S haplotypes, 28S GenBank accession numbers, and bibliographic sources.

Parasite species	Host species	Site	COI haplotype	Source	28S haplotype	28S G.B.A. no.
*Polystomoides asiaticus*	*Cuora amboinensis*	Oral cav.	H7	[45]	Hnuc12 (H7)	FM992703
						
*Uteropolystomoides nelsoni*	*Pseudemys nelsoni*	Oral cav.	H43	[45]	Hnuc20 (H43)	KR856156
*Polystomoides ocellatum*	*Emys orbicularis*	Oral cav.	H67, H89	[12]	Hnuc1 (H89)	OP795805
Polystomoides oris*	*Chrysemys picta*	Oral cav.	H11, H12, H14, H15, H33, H34	[12, 45]	Hnuc6 (H11, H12, H14, H33	FM992705
	*E. orbicularis*	Oral cav.	H63, H97 to H99, H115		H34, H63, H97 to H99, H115)	
	*Mauremys leprosa*	Oral cav.				
Polystomoides rohdei**	*Trachemys dorbigni*	Oral cav.	H52	[12, 28, 45]	Hnuc19 (H52)	OP795806
*Polystomoides scriptanus*	*T. scripta*	Oral cav.	H35, H50, H51, H107, H118	[12, 13]	Hnuc21 (H35, H51, H107)	
*Polystomoides soredensis*	*T. scripta*	Oral cav.	H16, H47, H49, H64, H77	[12]	Hnuc7 (H16, H49)	KR856154
*Polystomoides tunisiensis*	*M. leprosa*	Oral cav.	H25 to H30, H59, H65, H69	[12, 28, 45]	Hnuc5 (H25, H26, H59, H65	KR856155
			H78, H82, H85, H105, H106		H69, H78, H82, H85, H105, H106)	
*Polystomoides* sp1	*E. orbicularis*	Oral cav.	H66, H68, H95	[12]	Hnuc3 (H66, H68, H95)	OP795807
*Neopolystoma* sp.	*Apalone spinifera*	Oral cav.	H1, H2	[45]	Hnuc18 (H1, H2)	KR856149
*Fornixtrema palpebrae*	*Pelodiscus sinensis*	Conj. sacs	H41	[45]	Hnuc27 (H41)	AF38205
*Polystomoidella whartoni*	*Kinosternon baurii*	Urin. bladder	H23	[45]	Hnuc38	MW029411
*Uropolystomoides malayi*	*C. amboinensis*	Urin. bladder	H8	[45]	Hnuc32 (H8)	FM992704

Abbreviations used. Oral cav. = Oral cavity; Conj. sacs = Conjunctival sacs; Urin. bladder = Urinary bladder. G.B.A. no. = GenBank Accession number.

* *Polystomoides oris* was sampled from *Chrysemys picta* in the wild and from *E. orbicularis* and *M. leprosa* in turtle enclosures of private farms.

** *Polystomoides rohdei* was mistakenly considered *P. coronatum* in Verneau et al. [45], Meyer et al. [28] and Héritier et al. [12].

The COI phylogenetic analysis was conducted on a data set comprising 64 haplotypes and 396 characters which was considered a single partition. A GTR + I + G model was selected following the Akaike Information Criterion (AIC) implemented in Modeltest 3.06 [35]. Six types of substitutions and invariable-gamma rates with four gamma rate categories were therefore applied. On the contrary, the 28S phylogenetic analysis was conducted on a data set comprising 15 haplotypes and 1,370 characters also considered as a single partition. A GTR + G model was selected following the AIC, with six types of substitutions and gamma rates with four gamma rate categories. The Bayesian analyses were run using MrBayes 3.04b [14], with four chains running for one million generations and sampled every 100 cycles. The Bayesian consensus trees were drawn after removing the first 1000 trees (10%) as the burn-in phase and viewed with TreeView version 1.6 [33].

Corrected pairwise distances were calculated for COI sequences using the Kimura 2-parameter model, while the total number of differences was estimated for partial 28S in MEGA version 7 [17]. Species delimitation was discussed in the light of the COI threshold defined for polystomes [12].

## Results

### Morphological delimitation of the clade grouping *Neopolystoma*, *Polystomoides*, and *Uteropolystomoides* spp.

**Figure 2:**
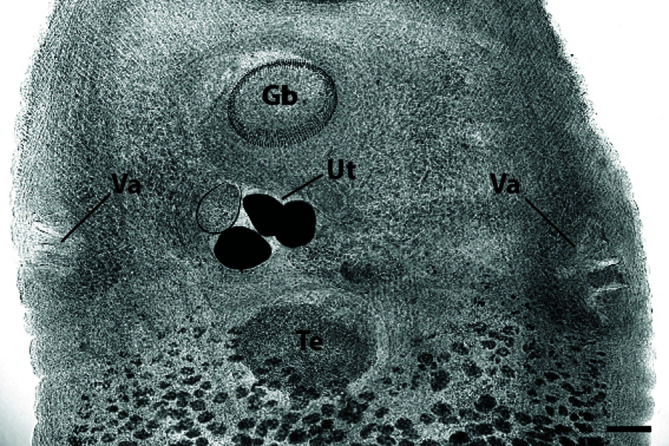
Micrograph of the reproductive system of *Polystomoides multifalx* (Stunkard, 1924). Abbreviations: Gb, genital bulb; Te, testis; Ut, Uterus with eggs; Va, vagina. Scale bar = 200 μm.

After examination of newly collected specimens, as well as types and paratypes of *Neopolystoma*, *Polystomoides*, and *Uteropolystomoides* spp. borrowed from museum collections, no obvious morphological character was evidenced supporting the clustering of these three genera into a clade with the exception of the vaginae that are peripheral (Fig. 2). Following a thorough study of all the drawings published in the literature for chelonian polystomes (see Morrison and Du Preez [30] for a review), this character is found in all species of the genera. It also characterizes all species of *Fornixtrema* and some polystome species of *Uropolystomoides* infecting specifically cryptodire turtles.

### Systematics of *Uteropolystomoides*, a monotypic genus infecting *Pseudemys* spp.

Measurements obtained from the 10 polystomes collected from *Pseudemys nelsoni* (Table 5, column 1) and the 10 collected from *P. concinna* (Table 5, column 2), showed an overlap indicating that all specimens belong to a single species. We therefore combined the measurements from the two polystome samples into a single set of data with their range, mean, and standard deviation (Table 5, column 3).

**Table 5:** Relative placement of some organs as % measurements from anterior end and average body measurements in micrometer for polystomes collected from *Pseudemys nelsoni* Carr, originally regarded as *Uteropolystomoides nelsoni* (Du Preez & Van Rooyen 2015) and from *Pseudemys concinna* (Le Conte), originally regarded as *Polystomoides stunkardi* Harwood, 1931. The fourth column combines measurements obtained from both samples. Measurements are presented as the range followed in parenthesis by the mean, standard deviation, and sample size.

Morphological characteristics	Sample collected from *Pseudemys nelsoni*	Sample collected from *Pseudemys concinna*	Combined set of data
Body length (BL)	4,730–7,745 (6,303 ± 965; 10)	5,408–10,691 (7,182 ± 1,653; 10)	4,730–10,691 (6,743 ± 1,392; 20)
Greatest width (GW)	1,761–2,865 (2,408 ± 373; 10)	1,859–3,058 (2,490 ± 404; 10)	1,761–3,058 (2,449 ± 381; 20)
Vaginal position from anterior as %	29–36% (32% ± 2%; 10)	28–38% (32% ± 3%; 10)	28–38% (32% ± 2%; 18)
BL / GW	2.2–3.9 (2.7 ± 0.5; 10)	2.3–3.6 (2.9 ± 0.4; 10)	2.2–3.9 (2.8 ± 0.5; 20)
Oral disk width	684–1,110 (927 ± 136; 10)	773–1,281 (997 ± 168; 10)	684–1,281 (962 ± 153; 20)
Pharynx length	417–676 (548 ± 77; 10)	455–661 (560 ± 61; 10)	417–676 (554 ± 68; 20)
Pharynx width	619–959 (756 ± 98; 10)	750–954 (822 ± 97; 10)	619–959 (783 ± 93; 20)
Haptor length (HL)	1,202–1,665 (1,423 ± 168; 10)	1,130–2,043 (1,496 ± 270; 10)	1,130–2,043 (1,459 ± 222; 20)
Haptor width	1,756–2,302 (2,070 ± 184; 10)	1,409–2,657 (1,982 ± 356; 10)	1,409–2,657 (2,026 ± 179; 20)
HL as % of BL	17–26 (23 ± 2.9; 10)	16–23 (21 ± 2.0; 10)	16–26% (22% ± 2.6; 20)
Haptoral sucker diameter	343–477 (419 ± 34; 51)	382–462 (418 ± 20; 53)	343–477 (419 ± 28; 104)
Ovary length	164–281 (213 ± 35; 9)	131–350 (258 ± 76; 7)	131–350 (233 ± 59; 16)
Ovary width	91–142 (112 ± 18; 9)	70–192 (139 ± 39; 7)	70–192 (124 ± 31; 16)
Egg length	137–263 (221 ± 49; 11)	241–269 (255 ± 13; 5)	137–269 (232 ± 43; 16)
Egg width	139–180 (160 ± 14; 11)	184–193 (189 ± 4; 5)	137–193 (169 ± 19; 16)
Number of eggs *in utero*	0–8 (3.4 ± 2.6; 10)	0–12 (3.8 ± 5; 10)	0–12 (3.6 ± 4.1; 20
Vagina length	360–860 (553 ± 124; 20)	353–722 (578 ± 109; 18)	353–860 (565 ± 156; 38)
Genital pore position from anterior as %	18–24% (22% ± 2%; 9)	18–24% (20% ± 2; 10)	18–24% (21% ± 2; 19)
Genital bulb diameter	438–847 (625 ± 130; 10)	498–781 (675 ± 88; 10)	438–847 (650 ± 111; 20)
Genital spines number	123–136 (130 ± 6; 7)	118–124 (120 ± 3; 7)	118–136 (125 ± 7; 14)
Genital spine length	96–98 (97 ± 1; 7)	83–98 (90 ± 5; 13)	83–98 (93 ± 3; 20)
Testis length	342–679 (471 ± 100; 10)	514–892 (628 ± 127; 9)	342–892 (545 ±136; 19)
Testis width	425–778 (617 ± 128; 10)	454–747 (450 ± 111; 9)	425–778 (632 ± 118; 19)
Hamulus handle length	105–145 (121 ± 15; 7)	108–175 (145 ± 22; 12)	105–175 (137 ± 22; 19)
Hamulus guard length	86–120 (104 ± 17; 5)	105–167 (131 ± 23,9; 7)	86–167 (121 ± 24; 12)
Hamulus hook length	60–78 (67 ± 7; 6)	59–86 (73; ± 9; 7)	59–86 (70 ± 9; 13)
Marginal hooklet 1 length	25–30 (28 ± 3; 14)	25–28 (28 ± 0.9; 11)	25–30 (28 ± 2; 25)
Marginal hooklet 2–8 length	25–29 (27 ± 3; 16)	24–29 (28 ± 1.3; 18)	24–29 (27 ± 3; 34)

**Figure 3:**
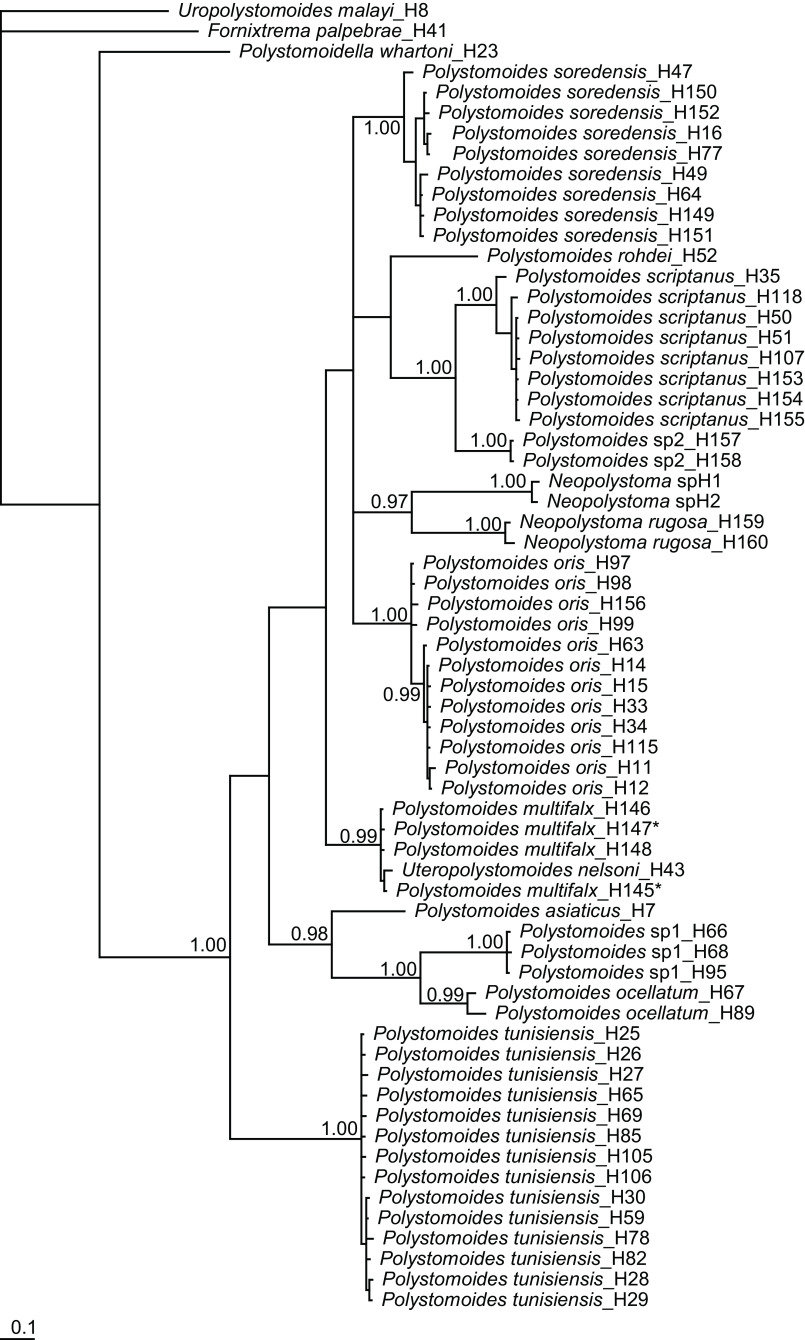
Bayesian tree inferred from the analysis of COI sequences. Numbers at nodes indicate Bayesian Posterior Probabilities (BPP). Only BPP ≫ 0.95 are indicated. Scale bar reflects expected changes per site. * designates haplotypes characterizing specimens of *Polystomoides multifalx* (Stunkard, 1924) that were, for some of them, collected from *Pseudemys concinna* (Le Conte), for the others, from *P. floridana* (Le Conte) (see Table 3 for more details).

**Figure 4:**
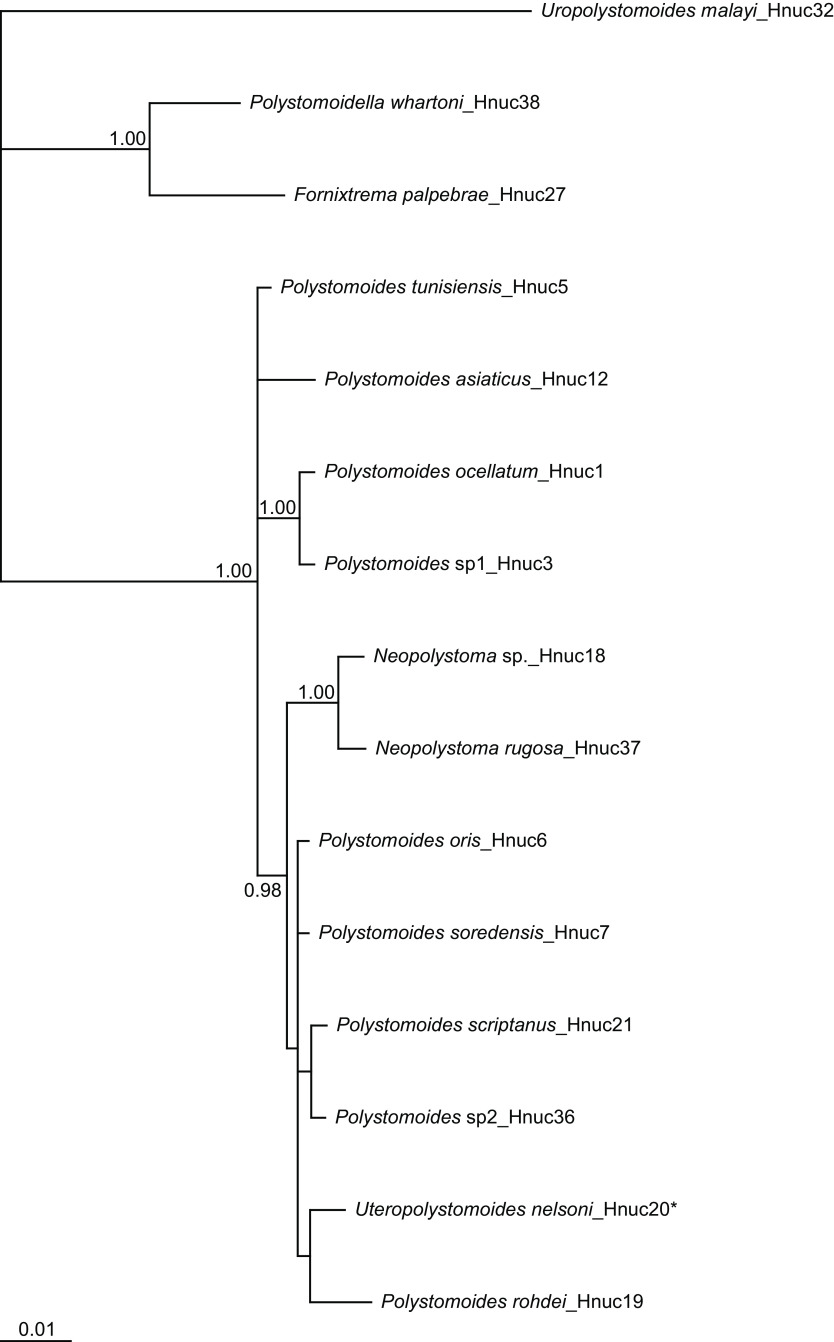
Bayesian tree inferred from the analysis of 28S sequences. Numbers at nodes indicate Bayesian Posterior Probabilities (BPP). Only BPP ≫ 0.95 are indicated. Scale bar reflects expected changes per site. * designates Hnuc20 haplotype that also characterizes specimens of *Polystomoides multifalx* (Stunkard, 1924) (see Table 3 for more details).

In the molecular study, we obtained 55 COI sequences including 16 new haplotypes (H145 to H160) and 13 28S sequences including two new haplotypes (Hnuc36 and Hnuc37). The resulting Bayesian consensus trees for COI and 28S are depicted in Figures 3 and 4, respectively. The COI tree shows 12 well-resolved lineages that each likely reflect a distinct parasite species. All COI haplotypes characterizing polystomes of *Pseudemys* spp. cluster in a single clade being strongly supported by Bayesian posterior probabilities. The 28S tree also shows 12 well-differentiated species, including *U. nelsoni* (Hnuc20) which shares the same haplotype with polystomes collected from *P. concinna* and *P. floridana* (see Table 3).

The Kimura-2 parameter distances for COI vary from 0.003 to 0.016 within polystomes collected from *Pseudemys nelsoni* (H43), *P. concinna* (H145 to H148), and *P. floridana* (H145, H147). The distance, however, varies from 0.110 to 0.180 between these parasites and their closest relatives. Additionally, a single 28S haplotype (Hnuc20) was reported for all polystomes collected from *Pseudemys* spp. That haplotype has seven mutations that differ from Hnuc6, Hnuc7, and Hnuc 21, which characterize *P. oris* Paul, 1938, *P. soredensis* Héritier, Verneau, Smith, Coetzer & Du Preez, 2018, and *P. scriptanus* Héritier, Verneau, Smith, Coetzer & Du Preez, 2018, respectively. On the contrary, two differences were observed in the 28S between *P. scriptanus* and *Polystomoides* sp2 of *Trachemys scripta* (Thunberg), between *P. oris* and *P. soredensis* and between *P. ocellatum* (Rudolphi, 1819) and *Polystomoides* sp1 of *Emys orbicularis* (Linnaeus). According to the threshold designed by Héritier et al. [12] within chelonian polystomes, that was set to 3.4% of COI genetic divergence, and to the high degree of 28S divergence between Hnuc20 and Hnuc19 (14 mutations), which characterizes the sister species of *U. nelsoni*, we suggest that all specimens collected from *Pseudemys* spp. belong to the same polystome species. This conclusion is strengthened by the existence of the same 28S haplotype within those polystomes.

## Discussion

### Systematics revision of *Polystomoides*

All the *Neopolystoma*, *Polystomoides*, and *Uteropolystomoides* spp. show similar morphology with vaginae that are peripheral and extend well beyond the intestine. Though this morphological characteristic is also found within *Fornixtrema* and some species of *Uropolystomoides*, *Fornixtrema* differs from these species by the shape of the egg and infection site, *i.e.* the conjunctival sacs, while *Uropolystomoides* differs by the shape of its first pair of hamuli. For these reasons, we propose the generic name *Polystomoides* for the entire clade after excluding *Uteropolystomoides* (see below). According to the principle of priority in the International Code of Zoological Nomenclature, article 23 [16], *Polystomoides* has priority over *Neopolystoma*. As a result, we reassign nine species, previously attributed to *Neopolystoma*, to *Polystomoide*s, and propose the following new combinations, namely *P. aspidonectis* (MacCallum, 1918) n. comb., *P. cayensis* (Du Preez, Badets, Héritier & Verneau, 2017) n. comb., *P. cyclovitellum* (Caballero, Zerecero & Grocott, 1956) n. comb., *P. domitilae* (Caballero, 1938) n. comb., *P. euzeti* (Combes & Ktari, 1976) n. comb., *P. exhamatum* (Ozaki, 1935) n. comb., *P. orbiculare* (Stunkard, 1916) n. comb., *P. rugosa* (MacCallum, 1918) n. comb., and *P. terrapenis* (Harwood, 1932) n. comb. It did not escape our attention that the type-species of *Neopolystoma*, *Neopolystoma orbiculare* (Stunkard, 1916), was nested in the clade (see Du Preez and Verneau [8]) but not the type-species of *Polystomoides*, *i.e. Polystomoides coronatum* (Leidy, 1888). Unfortunately*,* we could not sample the latter species because the identity of its type-host was fueled by ambiguity (see below). Nevertheless, in our estimation, based on the information available at present, *P. coronatum* should be attributed to this clade.

*Polystomoides* was originally created as a subgenus of *Polystoma* Zeder, 1800 by Ward [46] who designated *Polystoma coronatum* Leidy, 1888 as the type species. Ward (1917) based his subgenus chiefly on the presence of “a short uterus containing only a single egg”. Subsequently, *Polystomoides* was raised to the genus rank by Ozaki [32]. From 1939 until recently, the generic circumscription of *Polystomoides* was altered several times, and several species of *Polystomoides* were transferred to *Neopolystoma*, *Uropolystomoides*, *Uteropolystomoides*, and *Manotrema* on the basis of one character or a combination of characters [7, 36, 42, 43]. The type-species of *Polystomoides*, *P. coronatum*, was originally described by Leidy (1888) from a North American host turtle whose identity, “a common food terrapin”, was vague. Leidy [21] described it poorly and did not include any figures. *Polystomoides coronatum* was redescribed thoroughly and figured by Stunkard [39] from its type-specimen (No. USNM 1315426) and allegedly collected (quoting Stunkard) from *Emys palustris* Leidy, 1887 (now *Trachemys terrapen* (Bonnaterre, 1789)) and *Emys rugosa* Duméril & Bibron, 1835 (now *Trachemys decussata* (Gray, 1831)) (Stunkard, 1917). The genus *Polystomoides*, as redefined herein, groups only polystomes infecting either the oral cavity or the urinary bladder of cryptodires, with or without two pairs of small hamuli and some peripheral vaginae.

### *Uteropolystomoides*, a valid taxon?

*Uteropolystomoides,* as its generic name indicates, is characterized by the possession of a uterus containing a few eggs (up to 12 eggs in the present study). This feature was not found in *Polystomoides* or any other chelonian polystomes which possess an oötype where a single egg is often retained. The uterus is sacciform and pre-ovarian. Based on the phylogenetic relationship of polystomes infecting anurans, it was shown that *Polystoma*, the most widespread polystome genus, could represent a polyphyletic group, including a subgroup of species infecting specifically Asian frogs of India, China and Japan [1, 44]. By investigating the morphology of these species more in depth, Chaabane et al. [6] found some specific characters of these taxa that were used for describing a new genus, *i.e. Indopolystoma*, Chaabane, Verneau & Du Preez 2019 within the Polystomatidae. On the contrary, given the phylogenetic position of *Metapolystoma* which is nested within *Polystoma*, Bentz et al. [2] considered that *Metapolystoma* might be not valid. However, based on the morphology and life cycle of the monophyletic *Metapolystoma*, Landman et al. [20] concluded that this genus should be kept as a valid taxon within the Polystomatidae. Although we follow a cladistic approach in general to name groups and although *Uteropolystomoides* is nested in the *Polystomoides* clade, we propose to retain *Uteropolystomoides* as a valid genus based on its unique morphological characteristics.

### Revision of *Uteropolystomoides* outlines

*Polystomoides multifalx*, originally described as *Polystoma multifalx* Stunkard, 1924 from the pharyngeal region of *Pseudemys floridana* from central Florida (USA), was the first chelonian polystome known to have a huge genital bulb bearing numerous long spines in excess of 100 (120–124) [40]. Stunkard [40] mentioned that the number of genital spines of this species was three times greater than in any other known polystomes at the time. Based on samples from the mouth of *Pseudemys hieroglyphica* Boulenger (now *Pseudemys concinna*) from Oklahoma (USA), Harwood [10] distinguished *Polystomoides stunkardi* Harwood, 1931 from *P. multifalx* by the fewer genital spines, the smaller size of the genital bulb and testis, and the arrangement of haptoral suckers. From a morphological comparison between a set of specimens collected by Mr. Macintosh from *P. floridana* from southern Florida and vouchers of *P. stunkardi* from *P. concinna* from Oklahoma, Price [36] proposed the conspecificity of *P. stunkardi* with *P. multifalx*. However, Tinsley [42] concluded that *U. nelsoni*, *P. multifalx*, and *P. stunkardi* may form a coherent group of apparently related species. Based on morphological observations and measurements of samples collected from *P. concinna* and *P. nelsoni* (Table 5), we were unable to distinguish polystomes collected from both host species. Moreover, the genetic data indicated that polystome samples collected from the three distinct host species, namely *P. concinna*, *P. floridana,* and *P. nelsoni*, belong to the same polystome species. We therefore agree with Price [36], and consider that the specimens collected from *P. concinna* from the Ichetucknee River of Florida and those collected from *P. nelsoni* are conspecific with *P. multifalx*. We thus propose to consider a single species, namely *Uteropolystomoides multifalx* (Stunkard, 1924) n. comb. in the genus *Uteropolystomoides* and provide below a supplementary description for the new type species.

### Supplementary description of *Uteropolystomoides multifalx* n. comb.

Synonyms: *Polystoma multifalx* Stunkard, 1924; *Polystomoides multifalx* (Stunkard, 1924); *Polystoma stunkardi* Harwood, 1931; *Polystomoides stunkardi* (Harwood, 1931); *Polystomoides nelsoni* Du Preez & Van Rooyen, 2015; *Uteropolystomoides nelsoni* (Du Preez & Van Rooyen, 2015).

Taxonomy: Monogenea Bychowsky, 1937. Polystomatidae Gamble, 1896. Polystomoidinae Yamaguti, 1963.

Type-host and locality: *Pseudemys floridana* (Leconte, 1830) from central Florida, USA [40].

Other records: *Pseudemys concinna* (Leconte, 1830) from Oklahoma, USA [10]; *Pseudemys concinna* from southern Florida, USA [36] (based on the reported geographical distribution, this should be *P. floridana*); *Pseudemys concinna* from the Ichetucknee River in Ichetucknee Springs State Park of Florida, USA. *Pseudemys nelsoni* Carr, 1938 from Gainesville, Florida, USA.

Infection site: Oral cavity.

**Figure 5:**
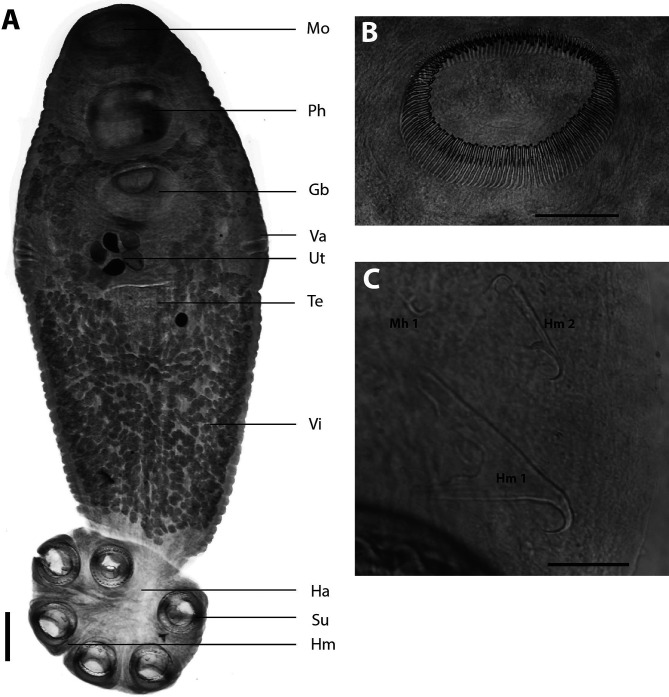
*Uteropolystomoides multifalx* n. comb. (Stunkard, 1924). A: Full parasite; B: Genital bulb with genital spines; C: Sclerotized haptoral hooks. Abbreviations: Gb, genital bulb; Ha, haptor; Hm, hamulus; Hm1, hamulus 1; Hm2, hamulus 2; Mh1, marginal hooklet 1; Mo, mouth; Ph, pharynx; Su, sucker; Te, testis; Ut, Uterus with eggs; Va, vagina; Vi, vitellarium. Scale bars: A = 500 μm; B = 200 μm; C = 50 μm.

Measurements (in micrometres): Body elongated and ellipsoid (Fig. 5A), dorsoventrally flat, 4730–10,691 (6743) long, 1761–3058 (2449) wide at vaginae, which is the widest point; position of vaginae 28–38% (32%) of total length measured from anterior end; body 2.2–3.9 (2.8) times longer than wide. Mouth surrounded by sub-ventral false oral sucker 684–1281 (962) in diameter. Pharynx 417–676 (554) long, 619–959 (783) wide. Intestine bifurcate with no diverticulae and no anastomoses extending full length of body proper, not entering the haptor and not confluent posteriorly. Posterior haptor 1130–2043 (1459) long, 1409–2657 (2026) wide, 16–26% (22%) of body length, bearing three pairs of cup-shaped haptoral suckers equal in diameter 343–477 (419), supported by a ring of well-developed skeletal elements. Ovary 131–350 (233) long, 70–192 (124) wide, elongate, not lobed, positioned pretesticular. Mehlis’ glands large, surrounding the base of the oötype. Uterus, spherical sac like, containing up to 12 ovoid, operculate eggs. Of the 19 specimens, five had no eggs, four had 1, one had 2, two had 3, two had 4, two had 6, one had 7, one had 8 and two had 12. Eggs 137–269 (232) long, 137–193 (169) wide. No intra-uterine development. Two lateral vaginae at the level of the ovary very prominent and big, 353–860 (565) long, bearing multiple marginal openings formed by branching vaginal canal. Vitellaria extended throughout most of body, except the ovary, uterus and genital bulb, and not entering the haptor. Stretching in between haptoral suckers, surrounding the female reproductive organs. Genito-intestinal canal, posterior to ovary. Testis 342–892 (545) long, 425–778 (632) wide, spherical, dense equatorial to post-equatorial. Vas deferens widens anteriorly to form the semen vesicle, narrowing towards genital bulb, opening in common genital opening. Genital pore opening ventral, directly posterior to intestinal ceca bifurcation, situated 18–24% (21%) of total length from most anterior point, genital bulb muscular, very big 438–847 (650) in diameter, surrounded by glandular cells, armed with a genital crown with 118–136 (125) genital spines (Fig. 5B), 83–98 (93) long. Two pairs of small hamuli (Fig. 5C) between posterior–most haptoral suckers with deep cut between handle and guard, handle 105–175 (137) long; guard 86–167 (121) long; hook 59–86 (70) long. Marginal hooklets placed as for other polystomes: pairs one and two between hamuli, marginal hooklet pairs three to five embedded in suckers, pairs six to eight between anterior suckers. Marginal hooklet pairs one 25–30 (28) long and hooklet pairs two to eight 24–29 (27) long.

## Conclusion

Following our investigations on morphological and molecular characters on the one hand, and based on the most updated phylogeny of polystomes infecting turtles on the other [8], we now consider nine genera within chelonian polystomes. According to the literature related to the taxonomy and systematics of polystomes, *Apaloneotrema* is a monotypic genus which infects the conjunctival sacs of cryptodire restricted to the Nearctic realm; *Aussietrema* comprises four species infecting the conjunctival sacs of pleurodires restricted to the Australian realm; *Fornixtrema* comprises seven species infecting the conjunctival sacs of cryptodires of the Indomalayan, Nearctic, Neotropical and Palearctic realms; *Manotrema* comprises three species infecting the urinary bladder of pleurodires restricted to the Neotropical realm; *Pleurodirotrema* comprises four species infecting the urinary bladder and the oral cavity of pleurodires restricted to the Australian realm; *Polystomoidella* comprises three species infecting the urinary bladder of cryptodires restricted to the Nearctic realm; *Polystomoides* comprises 29 species infecting the urinary bladder and the oral cavity of cryptodires distributed in the Nearctic, Neotropical and Palearctic realms; *Uropolystomoides* comprises 13 species infecting the urinary bladder of both pleurodires and cryptodires that are distributed in the Ethiopian and Australian realms, respectively on the one hand and in the Indomalayan realm on the other; *Uteropolystomoides* is a monotypic genus which infects the oral cavity of cryptodires restricted in the Nearctic realm. Regarding the distribution of polystome genera across chelonians and geographical areas, all genera with the exception of *Uropolystomoides* are restricted to a single group of turtles (pleurodires versus cryptodires), and usually found in a single or a few biogeographic realms. If future studies on the morphology of *Uropolystomoides* spp. split polystomes infecting pleurodires from those infecting cryptodires [7], it could demonstrate a correlation between historical biogeography of pleurodires and cryptodires and the diversification of polystomes. This deserves to be studied more in depth from a phylogeny including a larger sampling of species collected from all genera and ecozones.

## Conflict of interest

The authors declare that they have no conflict of interest.
